# ‘Irresponsible and a Disservice’: The integrity of social psychology turns on the free will dilemma

**DOI:** 10.1111/j.2044-8309.2011.02077.x

**Published:** 2013-06

**Authors:** James B Miles

## Abstract

Over the last few years, a number of works have been published asserting both the putative prosocial benefits of belief in free will and the possible dangers of disclosing doubts about the existence of free will. Although concerns have been raised over the disservice of keeping such doubts from the public, this does not highlight the full danger that is presented by social psychology's newly found interest in the ‘hard problem’ of human free will. Almost all of the work on free will published to date by social psychologists appears methodologically flawed, misrepresents the state of academic knowledge, and risks linking social psychology with the irrational.

Over the last few years, a number of works have been published suggesting both the putative prosocial benefits of belief in free will and the possible dangers of disclosing to the public doubts about the existence of free will. The names turning up most regularly as lead authors include Roy Baumeister and Tyler Stillman at Florida State, Kathleen Vohs at the University of Minnesotta, Jonathan Schooler and Azim Shariff at the University of British Columbia, and Daniel Wegner at Harvard. Baumeister has suggested that belief in free will reduces aggression and promotes selflessness (Baumeister, Masicampo, & DeWall, 2009). Vohs, Schooler, and Shariff have argued that disbelief in free will encourages cheating and undermines moral behaviour (Shariff, Schooler, & Vohs, 2008; Vohs & Schooler, 2008). Stillman, Baumeister, and Vohs have asserted that believers in free will make better employees (Stillman *et al.*, 2010). Stillman and Baumeister (2010) argue that belief in free will facilitates learning from emotional experiences while disbelief in free will reduces learning. And Wegner (2002) claims belief in free will is necessary for the sense of responsibility that underlies all morality.

In addition to asserting the putative prosocial benefits of belief in free will, such studies have often suggested that it is dangerous to disclose growing reservations about the existence of free will, and John Bargh at Yale has expressed disquiet about social psychologists potentially seeking to restrict such information. In an exchange with Roy Baumeister in the online blogs of *Psychology Today*, Bargh described what he called the ‘Vohs–Schooler–Baumeister’ concern to stay silent on doubts about the existence of free will as ‘irresponsible and a disservice … to the general public’ (Bargh, 2009). Bargh has furthermore noted that promoting the putative existence of free will in the absence of any objective evidence risks linking social psychology with the mystical.

John Bargh may be right to have drawn attention to the possibly ‘irresponsible’ writings of social psychologists, yet Bargh's work does not highlight the full danger to social psychology that is presented by this newly found interest in what philosophers have long called the ‘hard problem’ of human free will. For example, the Vohs–Schooler–Baumeister studies all appear to be linked by a fundamental methodological error that suggests that – irrespective of the separate question of suppressing knowledge – their putative findings may be spurious. Furthermore, the psychological literature appears almost wholly unaware that the possible existence of free will has already been disproved by logical philosophers. And it has long been recognized by certain scientists, legal theorists, and philosophers that far from having prosocial benefits, belief in free will acts to discriminate against the poor and racial minorities, may make justice impossible and even encourages contempt for and violence towards the weak. For example, research by the Joseph Rowntree Foundation has found that between 69% and 83% of Britons think that virtually everyone remains in poverty in Britain through choice, and that this belief in poverty-through-choice meant that respondents had become almost completely indifferent to the plight of the poor (Bamfield & Horton, 2009). Yet, although highlighting the dangers of failing to engage properly with the free will question, this paper will simultaneously suggest that psychologists may find the wider free will debates to have implications for multiple areas of current social psychology research including agency, intention, and forms of control.

## Do we have free will?

Table 1 effectively summarizes everything that has ever been written by academic philosophers, scientists, and theologians in defence of the notion of free will. Although others may define differently, this paper is concerned solely with the free will debate *as it relates to* freedom of choice, or the possibility that an individual could ever have done otherwise. Given this definition of free will as free choice, yet the need to refer to at least one tradition (compatibilism) that frequently uses the term differently, a single definition of free will becomes impossible. Notwithstanding how others use the term free will, however, this paper will always bring it back to an underlying stance on free choice. Furthermore, this paper is defining determinism as the recognition that everything that happens is determined by that which preceded it, as the understanding that all events in the quasi-classical – or non-quantum – universe have a cause. Determinism is contrasted with indeterminism that recognizes events with no cause, as in the quantum world. The following section may seem a little esoteric and would not normally find its way into a psychology journal. However, it is important to understand because now that social psychologists have started to join the free will debates the entire discipline may be judged by the quality of its contributions, both historic and future.

**Table 1:** Attitudes to free will

	Can free will and determinism co-exist?	Is determinism true (at the human level)?	Do we have free will?
1. Illusionism	No	Yes	No, but don't tell anyone
2. Compatibilism	‘Yes’	Yes	‘Yes’ (but not free choice)
3. Libertarianism	No	No	Yes, but we have no proof

The first grouping in Table 1 understands that free will does not exist but openly misleads the public over its non-existence. This is known as free will illusionism and occurs either through deliberately staying silent in the face of what theorists regard as public misperception (negative illusionism) or, more commonly, through overt deception (positive illusionism). The second grouping of writers understands that free will – defined as free choice – does not exist but redefines the meaning of the very words free will so as to mean other than free choice, and so that this grouping can continue to maintain that ‘free will’ exists even if free choice does not. This is known as compatibilism. These first two groupings thus understand that free choice does not exist, but either by commission or omission suppress this knowledge. The final grouping, metaphysical libertarianism, does still believe in the existence of actual free choice, though its writing on free will is today generally recognized – explicitly within mainstream science, and implicitly within mainstream analytical philosophy – to be a pure faith position, and an illogical one at that.

### Illusionism

Though compatibilism and libertarianism can be shown to have pedigrees back as far as the ancient Greeks – respectively, within Stoicism and Epicureanism – free will illusionism as a recognized tradition has a relatively recent history. There have been historical instances of the Christian Church expressing a desire to keep the truth on free will from the public, and, for example, the great 16th century Catholic theologian Erasmus wrote in 1524 that if there should prove to be no free will then, although an educated elite might be able to face such dangerous knowledge, the general public was too ‘weak’, too ‘ignorant’, and too ‘wicked’ to handle the knowledge (1524/1999, pp. 11–12). However, open deception on the issue of free will only entered philosophy a decade or so ago in the work of the philosopher Saul Smilansky. According to Smilansky, we ‘fragile … plants need to be defended from the chill of the ultimate perspective in the hothouse of illusion’ (2002, p. 501).

While Smilansky has picked up a handful of followers within philosophy, most philosophers are nervous about being caught out actively deceiving the public. Indeed, Smilansky is worried enough about his reception to claim that he has never argued for positive illusionism, for overt deception, and only for negative illusionism. If the public are naïve enough to believe in free will, let us not disabuse them of their mistake when such a mistake helps maintain the existing social order, Smilansky has written. Yet what is fascinating is that many scientists appear to hold none of the reservations of Smilansky, and indeed some call loudly in the technical press (both scientific and political) for not just negative illusionism but positive illusionism.

The quantum gravity theorist and best-selling science writer Paul Davies has called for the suppression of the scientific consensus that there is no free will. Free will, suggests Davies in a 2004 essay commissioned for the journal *Foreign Policy*, may be ‘a fiction worth maintaining’. Davies has called knowledge of the absence of free will one of the ‘world's most dangerous ideas’, even as he accepts that such knowledge has ‘more than a grain of truth’ to it (2004, pp. 36–37). Then there is Marvin Minsky, the celebrated cognitive theorist and co-founder of MIT's Artificial Intelligence Laboratory. In *The Society of Mind*, Minsky wrote: ‘According to the modern scientific view, there is simply no room at all for “freedom of the human will”. … Does this mean that we must embrace the modern scientific view and put aside the ancient myth of voluntary choice? No. We can't do that. … Such thoughts must be suppressed’ (1986, pp. 306–307). Another MIT figure within this camp is the Canadian cognitive theorist Steven Pinker. Pinker has admitted that free will is a deception society must be built on, although he prefers to call free will ‘an idealization’ rather than pejoratively calling it a deception (interviewed in Blume, 1998, p. 155). The Harvard psychologist and cognitive theorist Daniel Wegner is another apologist for the illusion of free will. In his 2002 *The Illusion of Conscious Will*, Wegner tells us that uncertainty over the existence of free will ‘makes everyone deeply uncomfortable’ and that in consequence, we should promote the politically expedient illusion of free will to the masses because ‘sometimes how things seem is more important than what they are’ (2002, pp. 336, 341). Or there is John Horgan– former chief writer at *Scientific American* – commenting as follows: ‘Science has made it increasingly clear (to me at least) that free will is an illusion. But – even more so than God – it is a glorious, absolutely necessary illusion’ (2000, p. 161). These are instances of what is now called free will illusionism.

### Compatibilism

Philosophical compatibilism dates back to the early Stoics and is the idea that free will is compatible with a fully deterministic universe, a universe where all current events are necessitated by past events, and that free will can co-exist with the understanding of humans as fully determined persons. (For humans, you can fit quantum indeterminism into a deterministic picture of actions because the quantum event may be uncaused but our resulting actions would be caused by the quantum event.) But how can free will be compatible with, at the human level, a fully determined universe? Because, for free will compatibilists, free will is simply redefined as being something other than freedom of choice, something other than freedom to have done otherwise, something other than freedom to have willed otherwise.

This is ultimately what all compatibilism boils down to; this is why Kant in 1788 called compatibilism a ‘wretched subterfuge … petty word-jugglery’, why William James in 1884 called it ‘a quagmire of evasion’ (James, 1884/1956, p. 149; Kant, 1788/1956, pp. 189–190). Within compatibilism, the general rule is that free will can be defined as anything, so long as it is never defined as freedom of choice, freedom for any actual individual to have done otherwise. So, for example, Susan Wolf has redefined free will to mean sanity (1989), whereas others have redefined it as freedom from constraint, as unpredictability, as acting in accordance with ‘reason’, and many, including the doyen of modern philosophical compatibilists Harry Frankfurt (1971/2003), as acting in harmony with your basic – causally determined – personality. The philosopher of science Dan Dennett (1984) has defined free will as mechanical self-control. Free will, according to Dennett, is possessed by yeast, chrysanthemums, and some plastic toys. Under Dennett's formulation were you to take your child's toy car, put in new batteries, and then set it to race away, it would not have free will; however, as soon as you turn your back and walk away from it never to return, you have blessed it with free will. In other words, free will has deliberately been defined so generously it becomes a meaningless term, a morally empty concept, a capacity we can share with both the Energizer Bunny and fungal infections.

There are some who continue to argue that the compatibilist project is a noble one, trying to steer a just course of limited human freedom through the murky waters of determinism, but I argue Dennett gives the lie to the suggestion that compatibilism is about advancing human understanding or justice. Dennett, one of the world's leading compatibilist scholars, tells us he finds facing up to the absence of free choice ‘almost too grim to contemplate’ (1984, p. 168). But what exactly does Dennett not wish to contemplate? As a free will theorist it is his job to contemplate the non-existence of free choice, so it cannot be his own contemplation he fears. No; what scares Dennett is not the fact of no free choice, it appears to be the risk of the public being given the chance to understand that fact. *Motive* becomes almost irrelevant; because, in a world without free will, ‘luck swallows everything’ (Strawson, 1998), one *effect* of denying the electorate such knowledge is to pretty much ensure that the lucky stay lucky while the unlucky remain unlucky.

Now some will object that others define differently and that, for example, there exist compatibilists who suggest that determinism is compatible with free choice. However, this paper is about free will defined as free choice, and if you are holding to the position that free choice is ever possible you are, fundamentally, a libertarian (or, at most, a ‘compatibilist-libertarian’). Yet you actually cannot be both a compatibilist and libertarian and remain internally coherent. An example may suffice. University of Illinois law professor Michael S. Moore is possibly the most influential retributivist scholar in Western legal theory, and Moore calls himself a compatibilist of the variant that holds determinism and free choice to be compatible. They are not. Moore notes that all we need to show to prove that an actor could have done otherwise is that he could have done otherwise ‘*if* he had chosen (or willed) to do otherwise’ (Moore, 1985, p. 1142). We need a *conditional* definition of the word ‘could’, says Moore, but to say an individual could have done otherwise *if* he or she had willed to have done otherwise is the logical equivalent of saying a dog would have a curly tail *if* it was a pig. Conditionality is not applicable here as no individual ever *could* have willed to have done otherwise in either a deterministic universe or an indeterministic universe. Two factors – and only two factors – determine human behaviour, being biology and experience, nature and nurture. At any particular moment, an individual could only have willed to have done otherwise if their biology or experiences to that date had been different; hence an individual could only have done otherwise if, in effect, they had happened to have been a different person. Person A is being judged *as person A* for not being person B, which sets up a paradox, and explains why you cannot legitimately conflate compatibilism and libertarianism. Similarly, this paper has ignored traditions such as semi-compatibilism and attitudinism because they are, at most, a repackaging of the traditions already discussed in Table 1. Hence although others may still object to the classifications within this paper, Table 1 both works and is useful when what you are trying to tease out is nothing more than *coherent* and *fundamental* positioning on free choice.

### Libertarianism

Metaphysical libertarian free will is the idea, the hope, that humans are *not* fully determined beings; that although we generally appear to exist within a deterministic universe (being a universe where all current and future events – at least from the point of view of human action – are necessitated by past ones), we somehow manage to break free from this causal chain. For libertarians an individual is truly the ultimate, the originating, cause of his or her actions, but libertarians never offer a coherent explanation for how ultimate origination can have a fundamental explanation; origination is posited but it is just supposed to remain a mystery. The author suggests that by number libertarians today account for no more than 20% of all philosophers, a small, and declining, band. So why is it that free choice is a faith position, an illusion, and not a scientifically or logically defensible platform?

Psychologists need to comprehend that the logic of the physical universe – indeed, any possible universe – rules out free choice, and irrespective of the indeterminism of quantum theory. Why? Well, because who a person happens to be from a moral point of view cannot possibly be under his or her control. To be responsible for how they act they would have to be responsible for how they are, and to be responsible for how they are they would have had to have created themselves, and no one can be the *causa sui*, the ultimate ‘cause of oneself’, because in order to do so, he or she would have to be in every sense his or her own parent, his or her own author. If you try to argue that a person has created himself or herself then you have to posit an earlier self that creates the later self, but then you note that the earlier self could not have created itself but must have been created by an earlier self, and you end up with an infinite regress of selves needed (Strawson, 1994). Yet, ultimately for libertarian free will you need an initial creator self, a ‘prime mover’ self, which is impossible to get to because how would it have come into existence?

Present-day libertarians tend to pin their hopes for free will on quantum mechanics, but the random chance of quantum theory has no connection whatsoever to the concept of ethical freedom; the freedom to *choose*, the freedom to *will*. Doing something because (hypothetically) a subatomic particle randomly moves inside your skull offers no more freedom than doing something because genes or culture dictate it. The quantum event may be uncaused, but your (hypothetical) resulting action would itself be caused by the quantum event. The action is therefore not uncaused, and it is most certainly not chosen or willed. Indeterminism cannot save free will for humankind, because if the mind is, at least in part, undetermined, then some things ‘just happen’ in it outside the laws of causation for which, by definition, nobody and nothing is responsible. An individual is not responsible if their actions are caused, because those actions were ultimately set in motion before they were even born. But an individual is also not responsible if some of their actions are uncaused, because those actions just came out of nowhere. To be freely choosing an individual would have to be free from both deterministic effects *and* indeterministic effects. Free from both A and not-A, as a logician would put it. To be freely choosing you cannot have A, but you cannot have not-A either; free choice requires something that cannot logically exist in this or any possible universe.

Even in philosophy, libertarianism is today generally viewed as a faith position, and a highly suspicious one at that. Peter van Inwagen is probably the leading modern libertarian philosopher, but in his book *An Essay on Free Will* he bases his belief in libertarian free will on the premiss that we ‘know’ we have moral responsibility for our actions. ‘To deny the free-will thesis is to deny the existence of moral responsibility, which would be absurd … therefore, we should reject determinism’, says van Inwagen, which is just begging the question, not proving the assertion (1983, p. 223). Even putting to one side van Inwagen's lack of engagement with the argument that free will is ruled out both by determinism *and* indeterminism, such an assertion is the logical equivalent of suggesting that we ‘know’ God exists so to reject theism would be ‘absurd’; it is empty circular reasoning where the conclusion is already built into the premiss. Another example of this faith position is given by the libertarian philosopher John Searle, probably the best-known academic advancing claims for supposed ‘gaps’ in human consciousness that might leave room for free will. In a 2000 paper Searle sought, over almost two-dozen pages, to prove free choice before admitting that, err, he could not prove it. ‘I have not tried to solve the problem of free will’, Searle wrote, after 19 pages of trying to solve the problem of free will. The flaw with each of his arguments, he admitted, is ‘to see how the consciousness of the system could give it a causal efficacy that is not deterministic’ (Searle, 2000, p. 21). Well, quite.

## The sins of social psychology

Now we understand the intellectual background; it is important to see where the current social psychology research fits in.

### The irrationality of social psychology

Almost all psychologists today writing on free will are libertarians, actually appearing to believe that the existence of ‘genuine … free will remains scientifically in question’ (Vohs & Schooler, 2008, pp. 53–54). In a similar vein we read: ‘such studies fail to put the proverbial nail in the coffin of free will’, because ‘an absence of evidence is not necessarily evidence of an absence’ (Shariff *et al.*, 2008, pp. 185, 195). Baumeister too is a libertarian: ‘To me, the essence of the idea of free will is that people actually make choices. … To determinists, choice is illusion. … They might be right. I am skeptical’ (Baumeister, 2009), and ‘the impossibility of free will cannot be proven either empirically or conceptually’ (Baumeister *et al.*, 2009, p. 260). Yet even John Bargh fails to state that free will has been proven to be an illusion, as when he agrees with Baumeister that we should be careful to present conclusions on the illusory nature of free will ‘as theory and not as established fact’ (Bargh, 2009).

It is incorrect to claim that the impossibility of free will has not been proven conceptually. It is true that *science* cannot prove the non-existence of free choice, because science is the search for what is, and not what is not, and similarly science cannot disprove the existence of gods, unicorns, or the Easter Bunny. But *logic*, which deals only in right and wrong answers, certainly can disprove the existence of free choice. We saw earlier that to be freely choosing an individual would have to be free from both deterministic effects *and* indeterministic effects. Free from both A and not-A, as we put it. To be freely choosing you cannot have A, but you cannot have not-A either; free choice requires something that cannot logically exist in this or any possible universe. Most of the great intellectual names of the pre- and post-enlightenment openly derided belief in free choice, including Hobbes, Spinoza, Voltaire, Darwin, and Einstein, and they did so *because* logic ruled out even its hypothetical existence. Nietzsche went so far as to call the idea of free choice ‘a kind of logical rape’ (1886/1990, p. 50).

### Social psychology as pseudo-science, and the moral ‘Hardness’ of social psychologists

The libertarians Shariff and Schooler admitted above that there is ‘an absence of evidence’ for free will. Note that they are not saying there is *limited* evidence, they are saying there is *no* objective evidence underlying the subjective libertarian faith in free will. Given this lack of proof despite centuries of investigation libertarians must surely admit that the existence of free choice remains, at the very least, highly speculative. The philosopher Richard Double (2002) has pointed out that few libertarians even purport to be able to provide objective proof that persons actually make free choices, but has then asked how much epistemic justification a libertarian should need before starting to claim people make free choices, with all the attendant contempt, loathing, blame, cruelty, revenge, and retribution this normally entails. (As we shall see below, belief in free will is heavily implicated in both Western contempt for the poor and the existence of extreme violence in the penal system.) Double has suggested libertarians have a moral duty to keep silent unless their suggestions are at least 50% likely to be true. He has called this the moral ‘hardness’ of libertarians; their unwavering faith in the righteousness of their worldview no matter the lack of any objective evidence and the pain caused to others. As Double puts it, ‘fallibilism about one's views is a desirable quality in general, but it is morally obligatory when dogmatism has potentially harmful repercussions for persons’ (2002, p. 231). It should furthermore be noted that asserting the existence of something for which there is zero objective evidence is widely viewed as a form of pseudo-science.

To consider for a moment, Western law recognizes that the penal system is so harmful to the existing life and future opportunities of persons that to convict requires evidence beyond a reasonable doubt. Yet libertarians provide *no objective evidence whatsoever* for the existence of free will, and therefore no apparent justification for the mass poverty and brutal punishments that belief in libertarian free will often brings with it. The leading legal theorist Stephen J. Morse freely admits that harsh prison conditions and execution are only morally tolerable where the presumption of free choice exists (1976a; 2004). Social psychology cannot claim to be ignorant of the increased hurt and even death that professing free will can bring because Jasmine Carey, a graduate student of Shariff and Schooler, has provided empirical evidence that belief in free will leads to harsher and more brutal punishment (Carey, 2009), while Baumeister himself has admitted that reductions in the capacity for free choice ‘constitute valid reasons for reduced punishment’ (Baumeister *et al.*, 2009, p. 260). There are very real costs to being wrong in this debate, and the leading libertarian philosopher Robert Kane (1996)– editor of *The Oxford Handbook of Free Will* – has distanced himself from his fellow libertarians by arguing that the inability to prove actual free choice obliges us to soften common formulations of blame.

Shariff, Vohs, and Schooler tell us that we should be prepared to ‘stake our very lives on the introspective certainty’ that we are conscious, but ‘perhaps none of us would be prepared to do the same for free will’ (Shariff *et al.*, 2008, p. 190). Yet surely social psychology risks appearing far too ready to stake *other* people's lives on the ‘introspective certainty’ of free will?

### The immorality of social psychology

And what of the social and moral downsides to belief in free will? In June 2009, the Joseph Rowntree Foundation published research showing that up to 83% of Britons think that ‘virtually everyone’ remains in poverty in Britain not as the result of social misfortune or biological handicap but through *choice* (Bamfield & Horton, 2009, p. 23; 69% of those surveyed agreed with the statement and an additional 14% were unsure but did not disagree.) Because of their belief in the fairness of ‘deserved inequalities’, such respondents were discovered to have become almost completely unconcerned with the idea of promoting greater equality while at the same time asserting that Britain was a beacon of fairness that offered opportunities for all. According to the Joseph Rowntree Foundation report, there was a ‘clear sense’ across all of the groups surveyed that an individual's situation ‘is largely of his or her own making’. Anyone can make it if they ‘really try’, the great majority of participants asserted (Bamfield & Horton, 2009, pp. 23–24). The 2010 *British Social Attitudes Report* also found that there has been a fall in those supporting any form of wealth redistribution, from 51% in 1994 to 38% in 2010; sympathy was now limited to those who did not ‘choose’ to live in poverty (National Centre for Social Research, 2010). Citing this second report, B.B.C. Radio 4's *Analysis* interviewed politicians and commentators to highlight a tendency within the welfare debates to distinguish between those who are poor through ill luck and those who are poor ‘because of personal choices’ (Bowlby, 2010).

Thomas Halper showed in a 1973 *Polity* essay that attitudes to poverty – including our corresponding compassion towards the poor or lack thereof – have always depended upon our views of personal merit, and choice in one's position, but Halper’s most worrying conclusion comes when he describes how the main reason for the great longevity and influence of the notorious concept of the deserving versus undeserving poor ‘was its profound legitimating power’ (1973, p. 76). When some are identified as deserving their station through choice rather than luck or accident it becomes not only an excuse not to do anything to help them but also, even more malignantly, is given as proof that such a society is just. The social historian Steve Hindle (2004) likewise argues that the pre-19th century growth in compassion for (some of) the poor appears to have been matched by the growing reliance on belief in choice as producing the varied stations of poverty, while Haggard (2000) actually suggests the development of the early British welfare state was only possible due to a declining belief in poverty through choice. In 2009, Jasmine Carey at the University of British Columbia surveyed over 250 undergraduates and found that those who believe in unrestrained free will were significantly more likely to believe in a just world for themselves and others, with just world here defined solely as the tendency ‘to believe that people deserve the things that happen to them’. As Carey noted, ‘the responsibility of free will is necessary for belief in a just world’ (2009, pp. 8, 20). Free will becomes the legitimating excuse that is used to ignore the plight of the most unfortunate, as the world is not now examined to *see* if it is just but instead is simply *assumed* to be just.

### The politicization of social psychology

Stephen J. Morse is Ferdinand Wakeman Hubbell Professor of Law at the University of Pennsylvania, and has for three decades been the doyen of legal compatibilists, the doyen of those who argue the law must be blind to questions of freedom of choice. And why might some legal theorists wish to keep alive the idea of free choice? Well, according to Morse, if society were to admit that no one chooses their position in life it would necessitate ‘the wholesale reform of society’ and ‘massive social reforms’ (1976a, pp. 1257, 1261), and ‘a massive redistribution of wealth’ and ‘social engineering … inconsistent with our system’ (1976b, p. 1276). Morse tells us that those who wish to query the law's position on free choice are motivated by the desire for ‘a truly egalitarian democracy’, and wish social reforms ‘not compatible with a [economically] libertarian and capitalist society’ (1976a, pp. 1258, 1261). All of which leads the legal theorist Anders Kaye at the Thomas Jefferson School of Law to note that any legal approach, such as compatibilism, which plays down the fact that choice is lacking is more conducive to the use of state violence against the disadvantaged, and that the disadvantaged tend to be ‘disproportionately – though not exclusively – people of color. … This is also the group with the most reason to criticize and challenge the social order’ (2007, pp. 418–419). Kaye has called this the ‘secret politics’ of the compatibilist criminal law. Free will is a belief that hurts the poor, acts to advantage the rich and powerful, and discriminates against the coloured, and it is crass not to recognize the unfair advantage the myth of free will may be giving to some segments of society, and very much at the expense of others.

### The methodological error of social psychology

Vohs, Schooler, and Shariff claim that believers in free will are more moral and less likely to cheat, while Baumeister claims believers in free will, and disbelievers in determinism, are more prosocial, less aggressive and less selfish, and more likely to work hard. But let us look a little more closely at these studies. Baumeister writes that the Vohs and Schooler study ‘proposed that disbelief in free will serves as a subtle cue that exerting volition is futile and thereby gives people permission not to bother’ (Baumeister *et al.*, 2009, p. 261), and, referring to his own studies, that ‘volition and self-control require the person to expend energy … apparently disbelief in free will subtly reduces people's willingness to expend that energy’ (p. 267). Stillman *et al.* (2010, p. 44) state that ‘a deterministic view … suggests that efforts do not matter. … Thus, one can predict that determinists … would not put forth the effort required to perform one's job well’. Vohs and Schooler ask why they got their results: ‘does the belief that forces outside the self determine behavior drain the motivation to resist the temptation to cheat, inducing a “why bother?” mentality?’ (2008, p. 54). But on this evidence, none of these studies have actually been investigating the effects of *disbelief in free will*. Instead the Vohs, Schooler, and Baumeister camps appear to have been subtly conditioning their interviewees to demonstrate the effects of *belief in fatalism*. Confusing determinism and fatalism is lack of knowledge of the history of the free will debates; almost two and a half thousand years ago, the Stoics showed that an understanding of determinism should never imply fatalism.

Determinism means that every action in the quasi-classical universe has a cause. In contrast, fatalism is a resignation to events that suggests that as everything is determined it is pointless to act because of a belief that no matter what one does one's future will not change. The Stoics called this the ‘lazy’ or ‘idle’ argument, and Cicero said that Chrysippus (ca. 270–207 BCE, Before Common Era) criticized the fatalist argument by showing that determinism left plenty of room for taking action. Fatalists used to argue over the point of calling the doctor if you are ill, but Chrysippus showed that you will get better by calling the doctor; it is just that the act of calling the doctor is itself also part of the chain of cause and effect. Where there is no free will, actions and efforts have effects and change the outcome from what it would have been if the effort had not been made; it is just that the outcome can still be defined in terms of prior causes.

It is worrying that many social psychology papers appear to mistake fatalism for determinism because dogmatic Christian writers have been guilty of this ‘lazy’ argument since Origen. However, even big-name libertarian philosophers still indulge in this palpable error. In a 2001 paper, John Searle states that even a determinist must act on the supposition of freedom; his argument is that a determinist in a restaurant would not be able to refuse to exercise his or her free will. ‘So if you say to the waiter, “Look, I’m a determinist … I’ll just wait and see what I order”, that refusal to exercise free will is only intelligible’ if you take it as an exercise of free will (2001, p. 494). But a determinist will make as many decisions as a libertarian, it is just that he or she will recognize their decisions as fully determined. The determinist will still select the fish over the wood pigeon, he or she just will not cast the runes seeking instruction, offer up a quick prayer for guidance, or invoke this as proof of either God or free will. No determinist has ever refused to make a decision; that is fatalism, not determinism. Abraham Lincoln is one of the best-known American determinists, who, although he daily rejected the idea of free will (Guelzo, 1997), made multiple decisions that define contemporary America. As a determinist understands that all effects need causes, and that all change only comes about through effort, a determinist is perhaps *more* likely to strive harder than most. I make the effort to write this paper *in order to have an effect*; the effect of, at the minimum, ensuring social psychologists become better informed about the free will debates. Vohs, Schooler, and Baumeister appear to have to date been testing the effect of belief in fatalism, not the effect of disbelief in free will.

## ‘Sometimes How Things Seem is More Important Than What They Are’

Now it might just be suggested that this paper was philosophy trying to close down legitimate discussion within psychology, whereas one of its actual goals is to get psychologists to force open the discussion, including within science and philosophy. On the issue of free will, the author believes that the record of a great number of contemporary philosophers, legal theorists, and senior scientists has been dismal. Although readers may yet feel that the scientific community has little to apologize for as the scientists listed earlier as illusionists are no longer the top names in any field, they were only selected because they use the most revealing language. Others who have used a scientific platform to defend the non-scientific concept of free will include biologists of the influence of Richard Dawkins, FRS, and the father of sociobiology Edward O. Wilson, ForMemRS (Dawkins, 1982, p. 11; Wilson, 1978, pp. 71–77), and physicists of the international standing of Roger Penrose, FRS, Stephen Hawking, FRS, and the string theorist Brian Greene (Greene, 2004, pp. 455–458; Hameroff and Penrose, 1995; Hawking & Mlodinow, 2010, p. 178), while their fellow scientists have not stopped them from doing so. Yet why should psychologists, *qua* psychologists, have an interest in correcting philosophers and scientists? Writing from outside the discipline, social psychology has always seemed particularly valuable where it has made us face the truths about ourselves that we would rather not know, and the absence of free choice may be the ultimate in self-knowledge that makes us ‘deeply uncomfortable’ (Wegner, 2002). Furthermore, this paper would argue that the free will debates make reference to multiple areas of study already of interest to psychologists; including intergroup attributions and our tendency to scapegoat, how we know anything and the pathologies of accepted wisdom, the power and misuse of language, self-censorship and forms of control, and the inherent (ir)rationality of humankind, as well as much-debated topics such as agency, self-image, and intention. And although it might be objected that this essay reduces philosophy too far to just the tradition of analytical logic, even if true this acts not to belittle, for example, Bergson's immanentist approach but to ensure that the mechanical/analytical approach is at least internally coherent. Furthermore it might be said that reduction to the mechanical/analytical may make this debate irrelevant to ethical discussion for psychologists coming from alternative philosophies. But to dismiss as possibly irrelevant an attack on the analytic from within the analytic is to risk being insensitive to the real-world effects; for instance, the moral character of the current US criminal law is largely built upon the writings of those like M. S. Moore at University of Illinois, not Henri Bergson, and some of its characteristics are susceptible to strong analytical attack for precisely this reason.

Vohs and her co-authors have suggested that perhaps ‘denying free will simply provides the ultimate excuse to behave as one likes’, and that a scientifically backed repudiation of free will ‘may encourage debauched behavior’ as people disabused of the illusion ‘seem to, at least temporarily, abandon their moral code’ (Shariff *et al.*, 2008, pp. 182, 198; Vohs & Schooler, 2008, p. 54). Baumeister has claimed that belief in free will ‘supports honest, responsible, moral, helpful, non-aggressive, and otherwise prosocial behavior’ (Baumeister, 2009). Moral code? Honest, moral, and prosocial? The myth of free will has been linked to deceit for four hundred years now; the illusionist camp of Wegner is tied to, well, illusion; the compatibilist camp has been accused above of ‘wretched subterfuge’ and of being ‘a quagmire of evasion’; and the libertarian camp of Vohs and Baumeister is at least guilty of not examining too closely. We have seen evidence that the myth of free will is inextricably linked to contempt for the poor and the unlucky, that it undermines both legal and natural justice, and may even make a mockery of the conceit of Christian compassion for the poor and marginalized. According to Anders Kaye, the myth of free will even allows racial prejudice to find a home within the Western law. Honest, moral, and prosocial?

Of course, even if we were to begin to acknowledge the moral and intellectual downsides to the free will myth, this would not suggest that Vohs and Baumeister were right to claim that belief in free will may also have prosocial upsides. We have seen that Vohs and Baumeister appear as yet to have shown no such thing, because all they have been studying appears to have been the effect of an acceptance of fatalism, not disbelief in free will. Contrary to the claims made in social psychology journals, we appear to have seen no evidence to date that disabusing people of the myth of free choice encourages anti-social behaviour, yet significant evidence that the myth of free choice encourages immoral, unjust, prejudiced, and anti-intellectual behaviour. If nothing else, this paper should stand as an important corrective within the psychological literature on free will.

In Britain, and very likely America, 69% to 83% of the population may be using the myth of free will to excuse indifference to the poor. Free will may just be the primary excuse many use to legitimize a contempt for the poor that would exist independent of their professed belief in free will, but free will assertion nonetheless provides the ethical fig leaf for such contempt that would be far harder to rationalize (and therefore tolerate) without the myth of free will. Therefore, the myth of free will does not just excuse indifference to poverty, it creates and maintains much of that poverty in the first place. The illusion of free will makes us ‘who we are’, says Daniel Wegner at Harvard's Psychology Department (2002, p. 328), though Wegner says this without a hint of irony. Free choice cannot exist in this or any possible universe, and hence, says the philosopher Dan Dennett, we should just continue to blame people ‘within limits we take care not to examine too closely’, and despite the fact that any defence of free will invites the suspicion of ‘wishful thinking at best, hypocrisy at worst’ (1984, pp. 164, 169). Wegner echoes this turning-of-a-blind-eye sentiment when he says that ‘sometimes how things seem is more important than what they are’ (2002, p. 341), but how things seem is *never* more important than what they are for those, such as the poor and racial minorities, who are being discriminated against on this issue. It is time social psychologists stopped advocating illogic and the suppression of knowledge.
